# Evaluation of the ALIBIRD mHealth Platform for Care of Patients With Lung Cancer: Prospective Pilot Study

**DOI:** 10.2196/69525

**Published:** 2026-02-11

**Authors:** Jose M Iniesta-Chamorro, Maria Sereno, Beatriz Garrido-Rubiales, Marta Villarino Sanz, Isabel Espinosa-Salinas, Sandra Falagán Martínez, Gustavo Rubio Romero, Juan Moreno-Rubio, Beatriz Tabarés, Yolanda Martín, Irene Hernández de Córdoba, Maria Morales Parga, Natalia Molinero, M Victoria Moreno-Arribas, Guillermo Reglero Rada, Ana Ramírez de Molina, Enrique J Gómez, Enrique Casado

**Affiliations:** 1 Biomedical Engineering and Telemedicine Centre, ETSI Telecomunicación, Center for Biomedical Technology, Universidad Politécnica de Madrid, Madrid, Spain; 2 Instituto de Investigación Hospital 12 de Octubre (imas12), Hospital Universitario 12 de Octubre, Madrid, Spain; 3 Clinical Oncology Group, IMDEA Nutrition, CEI UAM + CSIC, Madrid, Spain; 4 Medical Oncology Department, Infanta Sofía University Hospital, Infanta Sofía and Henares Hospitals Foundation for Biomedical Research and Innovation (FIIB HUIS HHEN), Paseo de Europa, 34, San Sebastián de los Reyes, Madrid, 28702, Spain, +34 911 914 000.; 5 GENYAL Platform, IMDEA Nutrition, CEI UAM + CSIC, Madrid, Spain; 6 Institute of Food Science Research (CIAL), Consejo Superior de Investigaciones Científicas (CSIC)–Universidad Autónoma de Madrid (UAM), Madrid, Spain; 7 Molecular Oncology Group, IMDEA Nutrition, CEI UAM + CSIC, Madrid, Spain; 8 Centro de Investigación Biomédica en Red de Bioingeniería, Biomateriales y Nanomedicina, (CIBER-BBN), Instituto de Salud Carlos III, Madrid, Spain

**Keywords:** mHealth, digital health, usability, patient monitoring, PROs, PROMs, lung cancer, precision nutrition, patient-reported outcome, patient-reported outcome measure, mobile health

## Abstract

**Background:**

Mobile health (mHealth) represents a promising instrument for optimizing symptom management and important lifestyle strategies that enhance self-care and the quality of health care for patients with cancer. The ALIBIRD mHealth platform is a digital health solution specifically designed for the telemonitoring of oncology patients, fostering patient empowerment and supporting clinical decision-making.

**Objective:**

The primary objective of this study was to evaluate the patient experience with the ALIBIRD platform. In addition, the study aimed to assess clinical outcomes, particularly in symptom management, nutritional status, and lifestyle, using patient-reported outcome measures (PROMs).

**Methods:**

The evaluation was conducted over a 30-week period in patients with advanced lung cancer receiving active treatment. Outcome variables included usability, patient experience, symptom burden, lifestyle behaviors (diet, physical activity, and sleep), nutritional status, PROMs, and system-generated clinical alerts. Through the mobile app, patients reported symptoms and completed integrated REDCap (Research Electronic Data Capture) questionnaires assessing lifestyle behaviors and PROMs, while receiving personalized recommendations informed by nutrigenetic and gut microbiota assessments. Daily activity and sleep data were automatically captured using the Fitbit Inspire wearable. Clinicians remotely monitored patient data using a web-based dashboard and performed clinical actions when required, including phone calls, therapeutic adjustments, referrals, and appointment rescheduling. Statistical analysis included descriptive summaries and pre-post comparisons of clinical and patient-reported outcomes.

**Results:**

Out of 20 patients recruited for the study, 14 completed the intervention. The System Usability Scale yielded a score of 90, indicating high usability. Among the 14 completers, adherence to scheduled questionnaires ranged from 94% to 100% for several instruments, and wearable-based monitoring ranged from 66% to 96% across visits. Overall, the ALIBIRD platform collected and processed 3589 patient-reported outcomes related to physical activity, 3468 related to sleep, 679 on-demand symptom entries, and 1524 completed questionnaires. Clinically, 143 alerts were resolved within an average of 2.05 days, resulting in 2 referrals to emergency rooms and 2 early detections of disease progressions. Furthermore, more than 2100 personalized recommendations contributed to a 21% (3/14 patients) increase in adherence to the Mediterranean diet and a 14% (2/14 patients) increase in moderate physical activity.

**Conclusions:**

The evaluation of the ALIBIRD implementation yielded promising results in that it facilitated the adoption of healthier lifestyle habits while enhancing health self-management among oncology patients. The ALIBIRD mHealth platform emerges as an effective digital health tool that enables closer monitoring of patients and thereby more informed clinical decision-making.

## Introduction

### Clinical Context and State of the Art

Cancer represents a significant health problem that, according to statistics from the GLOBOCAN 2022 database, has an incidence of nearly 2.5 million cases and causes 1.8 million deaths worldwide [1]. Among the different types, lung cancer (LC) is the most commonly diagnosed cancer globally (12.4% of total cases) and is also the leading cause of cancer-related deaths (18.7% of total cancer deaths). Effective management of disease and treatment-related symptoms, alongside the provision of supportive care, is essential for improving the quality of life (QoL) of patients with LC. Nevertheless, health care providers frequently underestimate the severity of symptoms during oncology consultations, a phenomenon attributed to demanding schedules, substantial workloads, and patients’ hesitance to articulate their concerns [2,3]. Oncologists often prioritize the identification and management of severe adverse events, thereby overlooking the comprehensive spectrum of symptoms that significantly impact patients’ QoL [4,5].

Consequently, patients with advanced lung cancer (aLC) experience multiple unmet needs, including complex symptom management, nutritional deterioration, and emotional distress, which are often insufficiently addressed by traditional care models. These models struggle to provide frequent, personalized follow-up, resulting in suboptimal QoL and potentially avoidable clinical events. Recent literature highlights the need for innovative, patient-centered strategies and hybrid care models that combine in-person and remote monitoring to deliver timely, tailored interventions [6,7]. Digital health solutions, particularly mobile health (mHealth) platforms, offer a promising approach by enabling continuous telemonitoring and supporting a multidisciplinary, multidimensional framework that integrates symptoms, lifestyle, nutrigenetics, and microbiota. Within this context, patient-reported outcomes (PROs) and their validated measures (patient-reported outcome measures [PROMs]) have become key instruments for capturing patients’ health status [8,9]. Their electronic implementation (electronic PROs) allows patients to self-report symptoms, lifestyle, and treatment-related information, enhancing self-care and adherence while enabling proactive clinical monitoring. This, in turn, contributes to more efficient health care delivery by reducing unplanned visits, emergency room utilization, and hospital admissions [10,11].

Various lifestyle factors, such as a healthy diet and physical activity, significantly influence cancer treatment and prognosis. In aLC, the high prevalence of malnutrition and risk of cachexia underscore the need for nutritional support and lifestyle interventions [12,13]. Personalized nutrition and lifestyle recommendations have emerged as promising strategies, allowing the design of tailored dietary plans based on clinical and genetic profiles [14]. Nutrigenetics can positively impact cancer treatment by genotyping processes such as lactose, gluten, alcohol, and tobacco metabolism, as well as physical activity tolerance and predisposition to conditions such as constipation or insomnia, thereby informing tailored supportive treatments [15]. Furthermore, the intestinal microbiome is implicated in cancer development, progression, and treatment response, with dysbiosis frequently observed in various malignancies [16,17].

eHealth and mHealth interventions have been increasingly implemented to support follow-up and improve QoL in patients with cancer [18,19]. These approaches build upon broader evidence from information and communication technologies in health care, which have been successfully applied across various medical fields. Such interventions have been associated with reductions in unplanned hospital visits and earlier detection of symptom deterioration [20,21], improvements in health-related quality of life and symptom self-management [22], and high patient adherence and acceptability in clinical use [23]. Evidence from oncology-specific applications reinforces this trend: studies have shown that patients are willing to adopt digital tools when recommended by clinicians and demonstrate sustained engagement and high acceptability in clinical use [7,23]. Complementary evidence from recent mHealth evaluations indicates that these interventions are generally well accepted, with high usability and sustained patient engagement in oncology settings [24-26].

Comparative studies further suggest that mHealth-based follow-up can achieve outcomes equivalent or superior to conventional care in aspects such as physical function, symptom control, and patient satisfaction, supporting the development of hybrid care models that combine in-person visits with telematic monitoring [19,27,28]. Moreover, emerging evidence points to the potential cost-effectiveness of these interventions, which may optimize resource allocation in oncology care [29]. These findings reinforce the potential of mHealth to support integrated, multidisciplinary care models capable of addressing the complex needs of patients with advanced disease, ultimately enhancing patient empowerment and enabling better clinical decision-making.

### Description of the ALIBIRD mHealth Platform

The ALIBIRD mHealth platform was developed within the ALIBIRD2020-CM program (S2018/BAA-4343)—a multidisciplinary consortium of 5 institutions from the Community of Madrid, Spain—aimed at designing and validating precision nutrition strategies for patients with cancer [30]. The development followed a user-centered and participatory design framework [31,32], actively involving patients, clinicians, and researchers throughout all stages [33,34]. Agile methodologies were applied [35,36], using iterative cycles of design, prototyping, and validation to ensure usability, clinical relevance, and alignment with user needs. The platform was developed and internally validated over approximately 18 months prior to the clinical study, following this iterative, user-centered process, for its subsequent evaluation in a pilot intervention.

The platform comprises three main components: (1) a mobile app for patients, (2) a web-based tool for health care professionals, and (3) a back-end infrastructure that includes the application programming interfaces, databases, notification services, wearable and REDCap (Research Electronic Data Capture) [37] integration, and a rule-based engine.

**Figure 1.**
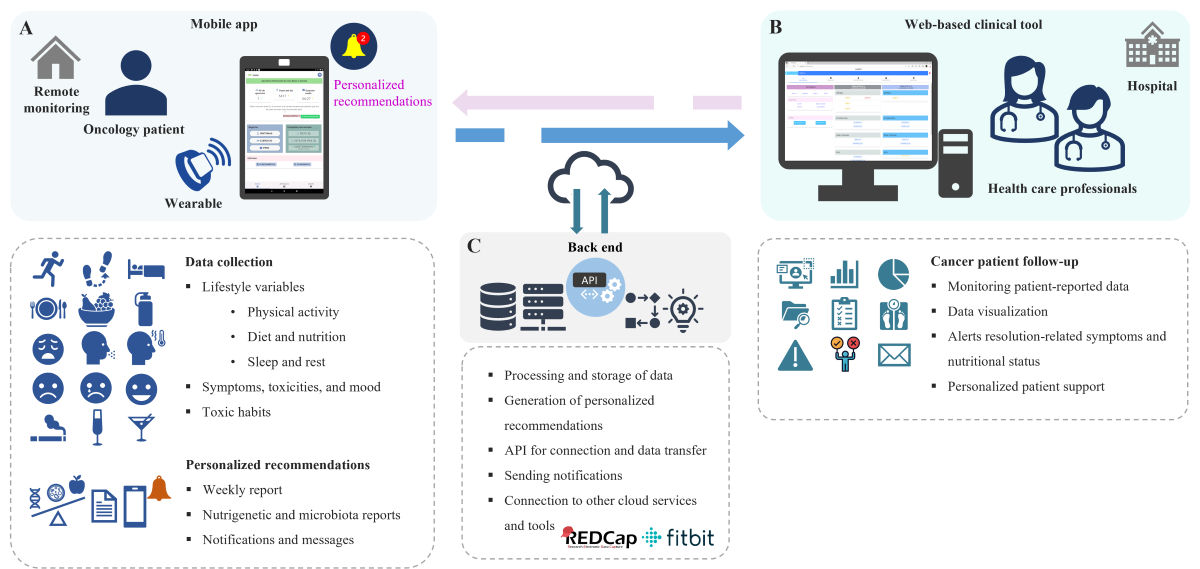
Overview of the ALIBIRD mobile health (mHealth) platform and the interactions and functionalities of its main components. The diagram illustrates (A) the patient mobile app, which collects lifestyle variables (physical activity, diet and nutrition, sleep and rest, and toxic habits), and patient-reported outcomes, including symptoms, toxicities, and mood, and delivers personalized recommendations (medication reminders, weekly reports, nutrigenetic and gut microbiota–based advice, and mobile notifications and messages); (B) the web-based clinical tool for health care professionals, which supports follow-up of patients with cancer through visualization of patient-reported data, resolution of symptom and nutritional alerts, and personalized patient contact; and (C) the back-end infrastructure, which enables secure processing and storage of data, generation of personalized recommendations and alerts, application programming interfaces for data connection and transfer, automated notifications, and integration with external cloud services and tools (eg, REDCap and Fitbit). API; application programming interface; REDCap: Research Electronic Data Capture.

The mobile app enables patients to report PROs and PROMs (symptoms, diet, and lifestyle) and to synchronize data from Fitbit devices to monitor physical activity and sleep. It provides personalized recommendations, nutrigenetic and gut microbiota reports, weekly progress summaries, and educational resources to promote self-management. The web-based tool allows health care professionals to remotely monitor patients’ symptoms, nutritional status, and lifestyle patterns, providing real-time access to alerts and reports. The back-end processes PROs, lifestyle data, and other clinical inputs to generate automated reminders, positive reinforcement messages, weekly summaries, and clinical alerts. It also incorporates baseline nutrigenetic analyses and gut microbiota profiling as structured inputs that support individualized nutritional guidance and lifestyle advice. These analyses are stored within the platform and contribute to tailoring recommendations delivered through the app and to the content available for clinicians during follow-up. Figure 1 illustrates the overall architecture and interactions of the ALIBIRD platform, including the patient mobile app (Figure 1A), the web-based clinical tool for health care professionals (Figure 1B), back-end services, and third-party platforms such as REDCap and Fitbit (Figure 1C).

Additional details of the rule engine and its logic, along with illustrative screenshots of the patient mobile app and the web-based clinical tool, are provided in Multimedia Appendix 1(Figures S1 and S2).

#### Personalized Recommendations and Alerts

The ALIBIRD platform includes a rule-based algorithm (rule engine) designed to generate automated recommendations and clinical alerts, informed by clinical guidelines, validated instruments, and expert consensus. The engine systematically analyzes PROs, lifestyle data, nutrigenetic information, gut microbiota findings, and other clinical inputs stored in the platform’s database. Within this framework, nutrigenetic and microbiota-derived insights are integrated directly into the decision logic to refine nutritional and lifestyle recommendations according to each patient’s biological profile.

Thresholds for dietary recommendations and nutritional risk (including weight management and malnutrition indicators) were derived from the Mediterranean Diet Adherence Screener (MEDAS), the Mediterranean Diet Serving Score, and the ESPEN practical guideline: Clinical Nutrition in Cancer [38-42]. Symptom severity was classified according to the Common Terminology Criteria for Adverse Events (CTCAE v5) [43], while recommendations related to physical activity and sleep patterns were aligned with widely accepted international standards, such as those from World Health Organization.

Four types of outputs are generated: (1) reminders to support adherence, (2) positive reinforcement messages to promote healthy habits, (3) weekly progress summaries, and (4) clinical alerts to notify health care professionals about adverse events or critical changes (eg, severe symptoms, reduced food intake, or significant weight loss). When an alert is triggered, clinicians can take timely actions, such as scheduling an appointment, adjusting treatment, or referring the patient to a specialist.

The development of the algorithm was informed by expert input from oncologists, nutritionists, and nursing professionals, with decision rules and thresholds being progressively refined to ensure clinically relevant and accurate outputs. Additional details of the rule engine, including monitored dimensions and a schematic overview of data processing and outputs, are provided in Multimedia Appendix 2 (Table S1 and Figure S1).

The primary objective of this study was to evaluate the usability and patient satisfaction with the mHealth ALIBIRD platform in patients with aLC. The secondary objective was to explore its clinical impact, including early detection of relevant events, resolution of alerts, and the promotion of healthier lifestyle habits.

## Methods

### Study Design

A single-arm, prospective, interventional study was conducted involving patients with advanced thoracic tumors, mainly non–small cell lung cancer (NSCLC), at the Infanta Sofía University Hospital in Madrid, Spain. The total study duration was 18 months, including a preparatory phase for study setup and patient recruitment, followed by a 30-week intervention period per patient. Patient recruitment occurred between November 2021 and January 2022, with consecutive enrollment as patients met eligibility criteria, resulting in a staggered but overlapping participation across the cohort.

#### Integration Into Clinical Workflow

In oncology, conventional patient follow-up is typically based on scheduled in-person visits spaced every few weeks, with limited patient-clinician interaction in between. In our study, we implemented a hybrid (mHealth-based) care pathway, integrating the ALIBIRD mobile app and clinical dashboard with usual care to support continuous self-management and remote monitoring.

**Figure 2.**
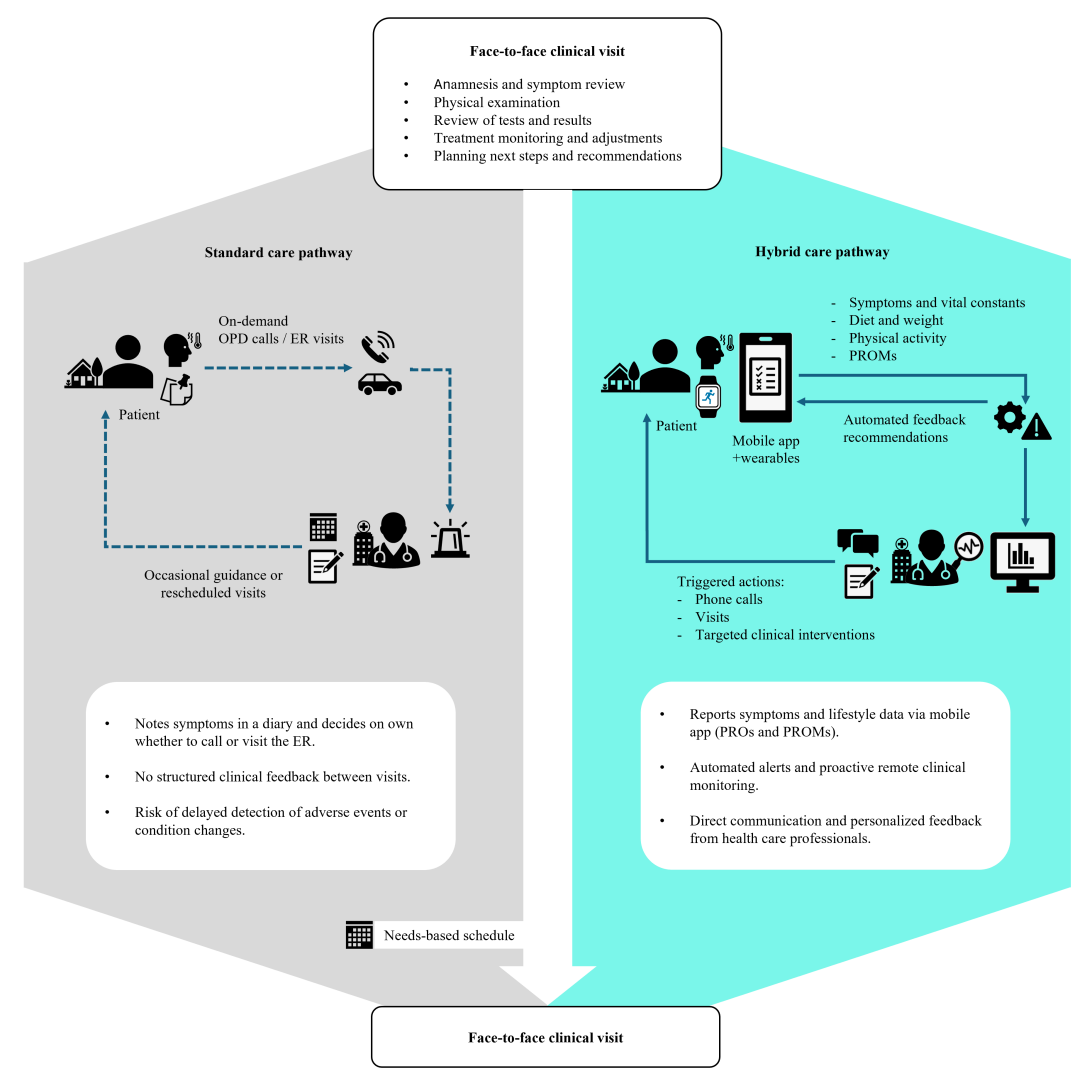
Integrating patient-reported outcomes and supportive oncology. The diagram compares the standard care pathway with the hybrid mHealth-supported pathway implemented in the study using the ALIBIRD platform. In the standard pathway, routine visits are scheduled and structured; however, clinical follow-up between visits relies on patient-initiated contact, leading to unstructured feedback and a higher risk of delayed recognition of adverse events. In contrast, the hybrid pathway incorporates continuous digital monitoring through the mobile app and wearable devices, enabling patients to report symptoms, lifestyle data, and patient-reported outcome measures. Automated algorithms generate alerts and personalized recommendations, prompting timely clinical actions such as phone calls, rescheduled visits, or targeted interventions. Continuous lines indicate structured digital follow-up, while dashed lines represent occasional or patient-initiated contact. ER: emergency department; OPD: outpatient department; PROMs: patient-reported outcome measures; PROs: patient-reported outcomes.

A multidisciplinary team of 5 clinicians (3 oncologists, 1 nutritionist, and 1 oncology nurse), supported by a data manager and a biomedical engineer, participated in patient monitoring and system oversight. Clinicians performed standard clinical visits (V1-V10) and used the dashboard to review patient-reported data, respond to alerts, and personalize interventions. The data manager conducted regular quality checks on data completeness, while the biomedical engineer ensured technical support and system performance (Figure 2).

#### Study Protocol

The primary objective was to evaluate the usability and patient satisfaction with the ALIBIRD platform in patients with aLC. The secondary objective was to explore the clinical impact of the system, including early detection of relevant events, resolution of alerts, and the promotion of healthier lifestyle habits. The intervention period lasted 30 weeks per patient and included 10 scheduled face-to-face visits (V1-V10), complemented by continuous remote monitoring through the ALIBIRD mobile app and a wearable device.

Eligible participants were consecutively identified by the study oncologists during routine outpatient visits for NSCLC. Once a patient met the inclusion criteria—histologically or cytologically confirmed NSCLC, aged 18 years or older, Eastern Cooperative Oncology Group performance status ≤2, ongoing systemic treatment, regular internet access, and sufficient digital literacy—and none of the exclusion criteria applied (eg, symptomatic brain metastases, neurological or psychiatric disorders, or inability to comply with study procedures), the oncologist presented the study and its objectives. Each candidate received an information sheet and provided written informed consent prior to enrollment.

Following enrollment, a biomedical engineer conducted individualized training sessions covering the purpose, scope, and functionalities of the ALIBIRD mobile app, as well as the proper use of the wearable device and the type and frequency of data to be recorded. Each participant received a Fitbit Inspire model wearable provided by the study team. Fitbit Inspire is an accelerometer-based device for monitoring daily steps, physical activity patterns, and sleep duration in adult populations. Continuous technical support was available throughout the study to address any issues or questions.

**Figure 3.**
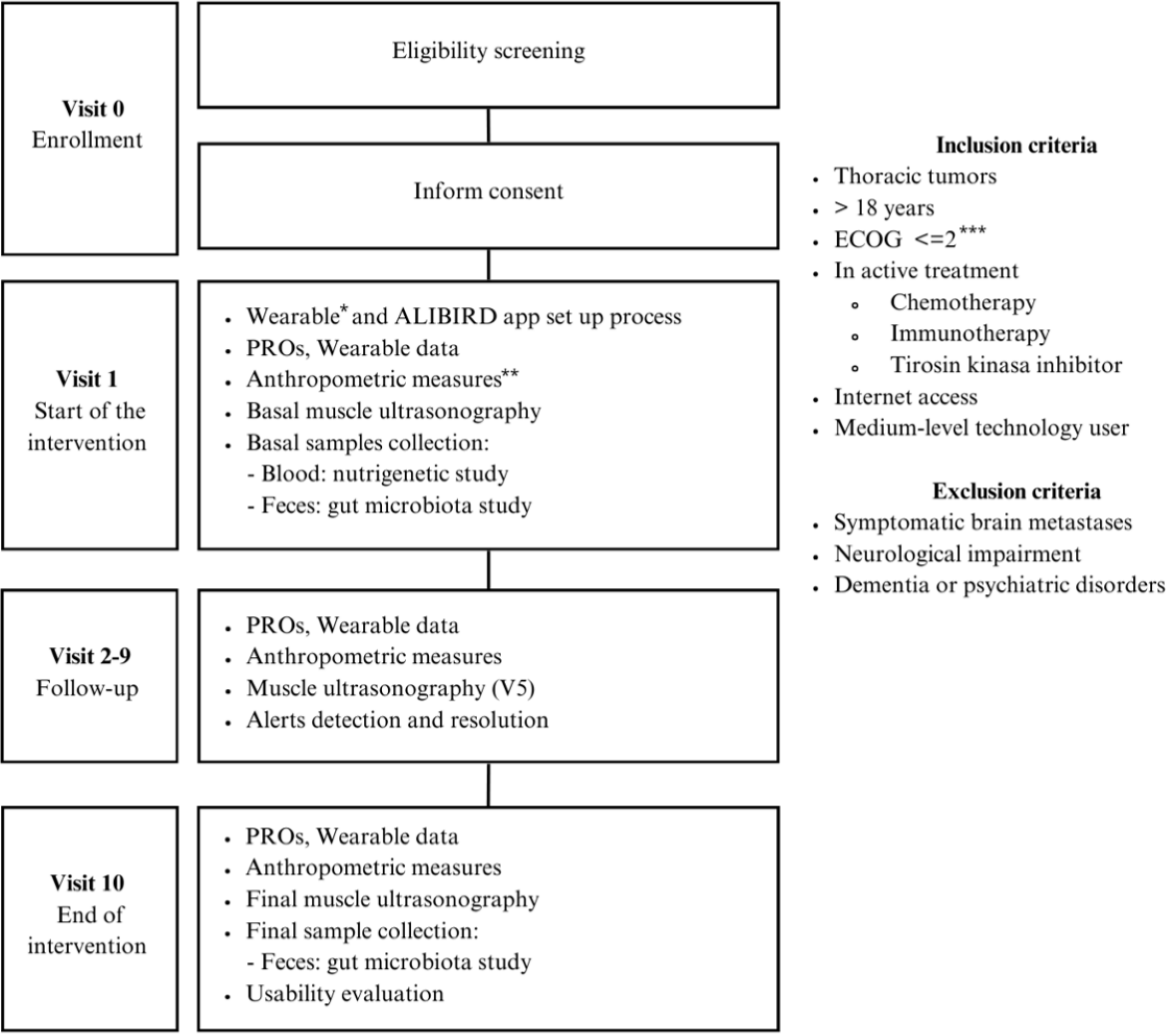
Schematic representation of the study protocol for the ALIBIRD prospective pilot intervention in patients with advanced lung cancer. The diagram summarizes enrollment procedures, inclusion and exclusion criteria, and the schedule of assessments across the 30-week study, including baseline setup of the mobile app and wearable device, patient-reported outcomes, wearable-derived activity and sleep data, anthropometric measurements, muscle ultrasonography examinations, and collection of samples for nutrigenetic and gut microbiota analyses. Follow-up visits (V2-V9) included continuous remote monitoring and alert resolution, while the final visit (**V10**) incorporated end-of-intervention assessments and usability evaluation. *Wearable device model: Fitbit Inspire. **Anthropometric measures include weight, BMI, and body composition. ***Eastern Cooperative Oncology Group is a widely used performance status scale in oncology. ECOG: Eastern Cooperative Oncology Group; PROs: patient-reported outcomes.

During the study, patients reported PROMs and lifestyle-related variables through the mobile app, while the wearable device automatically captured daily activity and sleep data. At each scheduled visit, oncologists conducted clinical assessments, and the nutritionist performed nutritional evaluations, including anthropometric measurements (weight, height, BMI, and body composition determined by bioimpedance and muscle ultrasonography). A research nurse was responsible for collecting biological samples—blood for nutrigenetic analyses and stool for gut microbiota characterization. A data manager supervised data quality and consistency across all data sources to ensure compliance with the study protocol and overall data integrity. Figure 3 illustrates the study flow, main intervention components, and inclusion and exclusion criteria.

All PROMs and patient-reported experience measures (PREMs), including the System Usability Scale (SUS), were implemented in REDCap and administered electronically through the ALIBIRD mobile app. Notifications were automatically sent via the app to remind participants when questionnaires were due, facilitating timely completion and high response rates. An overview of all data sources and assessment tools used in the study is provided in Table 1.

**Table 1.** Instruments and measures used for on-demand, continuous, and scheduled data capture during the 30-week ALIBIRD mHealth intervention in patients with advanced lung cancer. The table summarizes all variables and corresponding measurement instruments, including patient-reported outcomes, patient-reported outcome measures, wearable-derived data, nutritional assessments, sleep and physical activity questionnaires, toxicity reporting tools, and usability and experience measures, along with their specific capture frequency.

Acquisition instruments	Capture frequency
Symptoms	
Symptom diary (type and severity): *Symptoms were selected based on the most frequent events in aLC*^a^ *and classified into 3 severity levels: G1 (mild), G2 (moderate), and G3 (severe*)	On demand
European Organisation for Research and Treatment of Cancer Quality of Life Questionnaire—Lung Cancer 13 [44]: *Standard instrument for measuring the quality of life of patients with lung cancer; covers 13 typical symptoms and side effects from treatment. Higher scores indicate a high level of symptomatology*	V1-V10
Nutritional status	
Anthropometric measures: *Included weight, height, BMI, waist and hip circumference, and bioimpedance analysis*	V1-V10
Malnutrition diagnosis: The Global Leadership Initiative on Malnutrition criteria [45]: *Guidelines to diagnose malnutrition: at least 1 phenotypic and 1 etiologic criterion required*	V1 and V10
Muscle ultrasonography: *Ultrasonography of rectus femoris for sarcopenia assessment: area, y-axis (relaxation/contraction), x-axis*	V1, V5, and V10
Diet	
Mediterranean Diet Adherence Screener [42]: *14-item questionnaire to assess adherence to the Mediterranean diet*	V1 and V10
Dietary intake and appetite log: *Ad hoc questionnaire including daily number of meals, self-reported appetite, and food frequency for the last week (cereals, dairy, fruits, vegetables, oils, liquids, meats, fish, processed meats, etc*)	Weekly
Physical activity	
International Physical Activity Questionnaire short form [46]: *Standardized tool to classify activity levels into mild, moderate, and vigorous*	V1-V10
Fitbit: daily steps and physical activity sessions	Continuous capture
Sleep	
Pittsburgh Sleep Quality Index [47]: *Self-report questionnaire assessing sleep quality (eg, waking during the night and need for medication) and classifies it into 2 groups: poor or good quality*	V1, V5, and V10
Fitbit: Sleep sessions	Continuous capture
Toxicity	
Patient-Reported Outcomes version of the Common Terminology Criteria for Adverse Events [48]: *System developed by the National Cancer Institute to capture symptomatic adverse events in cancer trials*	V2-V10
Quality of life	
European Quality of Life-5 Dimensions 5 Levels [49]: *Generic self-assessed health-related quality-of-life measure across 5 dimensions (mobility, self-care, usual activities, pain/discomfort, and anxiety/depression); includes a visual analogue scale. Higher index scores indicate worse health*	V1-V10
Emotional status	
Hospital Anxiety and Depression Scale (HADS) [50]: *Self-report rating scale with 14 items on a 4-point Likert scale (0‐3). It measures anxiety (HADS-A) and depression (HADS-D) through 7 items each, with subscale scores ranging 0‐21 and a total score summing all items*	V1, V5, and V10
Perceived energy and emotional status: *Two ad hoc self-report questions: (1) overall mood during the past week (good, fair, and poor) and (2) perceived energy level (high, medium, and low*)	Weekly
Usability and satisfaction	
System Usability Scale [51]: *Widely used 10-item questionnaire; provides a 0‐100 usability score (higher is better*)	V10
Patient-reported experience measures: *3-item measure assessing motivation, satisfaction, and willingness to recommend the app*	V10

a aLC: advanced lung cancer.

The sample size of 20 patients was selected for convenience, considering the limited population of patients with NSCLC at our center and the sample sizes reported in similar studies [28,52-56].

#### Evaluation Methods

Evaluation of the ALIBIRD platform included the analysis of adherence and data completeness, usability and patient experience, and the clinical impact of the intervention. System use and adherence were assessed through the number and consistency of patient-reported entries via the mobile app, as well as data automatically collected by the wearable device. Usability was evaluated using the SUS [51,57], a 10-item questionnaire rated on a 5-point Likert scale, widely used for subjective assessment of system usability. Scores range from 0 to 100, with values above 68 considered above average and indicative of acceptable usability. In addition to patient evaluation, usability was also assessed among health care professionals using the same SUS questionnaire, administered electronically through REDCap via a web-based survey link at the end of the study.

Patient experience was measured through a 3-item PREM, which assessed motivation to adhere to healthy recommendations, overall satisfaction with the app and follow-up, and willingness to recommend the platform. The exact questions are summarized in Textbox 1.

Both the SUS and the PREMs were administered to patients at the end of the intervention (V10).

Clinical impact was explored by analyzing both alerts generated and the corresponding clinical actions, as well as PROs and lifestyle data, including symptoms and toxicities, QoL, diet adherence, physical activity, and sleep parameters.

Textbox 1. **Questions about patient experience with the mobile app: 2 of the questions used a 5-point Likert scale, while the third question used a Yes/No format to assess patients’ willingness to recommend the app.**

- “So far, the app has motivated me to follow guidelines to better control possible symptoms and adopt healthy lifestyle recommendations.” (Strongly disagree/Disagree/Neutral/Agree/Strongly agree)
- “How would you rate your level of satisfaction with the app and the follow-up you receive in the study?” (Completely dissatisfied/Dissatisfied/Somewhat satisfied/Satisfied/Completely satisfied)
- “Would you recommend an app with similar features and follow-up to someone you know who is in your situation?” (Yes/No)

#### Nutrigenetics and Gut Microbiota

Saliva samples were collected at baseline (V1) for nutrigenetic analysis, including genes related to metabolism, nutritional traits, and digestive predispositions. Results were based on single nucleotide polymorphisms (SNPs) and composite scores derived from multiple SNPs, with each SNP weighted according to the strength of scientific evidence (high=1, medium=0.5, and low=0.25). Favorable, intermediate, or unfavorable genotypes contributed to an overall score.

Fecal samples were collected at baseline (V1) and at the end of the intervention (V10) to assess gut microbiota composition and dysbiosis status. Key parameters were derived from the relative abundances of microbial groups associated with positive or negative health effects and the production of beneficial metabolites, such as short-chain fatty acids (Table S1 in Multimedia Appendix 3) [58-63].

The results of both analyses were used to generate personalized reports for patients and health care professionals, which were translated into tailored nutritional recommendations delivered through the ALIBIRD mobile app. Detailed protocols for DNA extraction, genotyping, and SNP scoring (nutrigenetics), as well as 16S rRNA sequencing, taxonomic assignment, and dysbiosis assessment (gut microbiota), are provided in Multimedia Appendix 3.

#### Data Analysis

Data were analyzed using descriptive statistics (frequencies, proportions, and means, SD), Shapiro-Wilk normality testing, and paired sample tests (*t* test or Wilcoxon) or McNemar test for categorical comparisons (V1 vs V10). The SUS score was calculated following the standard procedure (sum of item scores multiplied by 2.5 to obtain a 0‐100 scale), and PREM responses were summarized as frequencies and percentages. Data were exported from the system database and REDCap, preprocessed in Excel, and analyzed with IBM SPSS Statistics (version 29; IBM Corp).

### Ethical Considerations

The study was conducted in accordance with the principles of the Declaration of Helsinki (Fortaleza, Brazil, 2013, and subsequent revisions) and the International Council for Harmonisation Good Clinical Practice guidelines. The protocol and all related documentation were reviewed and approved by the Research Ethics Committee for Medicines of the Hospital Universitario La Paz, Madrid, Spain (approval code: HULP PI-4735). The study also complied with the Spanish Royal Decree 957/2020 of November 3, regulating observational studies with human-use medicines, and with the applicable regional regulations.

All participants received verbal and written information about the study’s objectives, procedures, and potential risks and benefits. Written informed consent was obtained from each participant prior to inclusion, in compliance with the ethical principles of autonomy and informed decision-making. Participants were informed of their right to withdraw at any time without providing justification and without any impact on their medical care.

The protection, processing, and analysis of study data were carried out in accordance with the General Data Protection Regulation (EU) 2016/679 and the Spanish Organic Law 3/2018 of December 5 on the Protection of Personal Data and Guarantee of Digital Rights, as well as Law 41/2002 of November 14 on Patient Autonomy and Rights and Obligations Regarding Clinical Information and Documentation. All data were pseudonymized and stored within a secure research data infrastructure encompassing both the REDCap and the ALIBIRD platform databases. These systems were hosted on secure servers and included encrypted data transfer, user authentication, and activity logging. Access to identifiable data was restricted to authorized study personnel only. No financial or material compensation was provided to participants for their participation in this study.

## Results

### Principal Results

The analysis focused on patient adherence, usability, and clinical impact of the ALIBIRD mHealth platform. Results are organized to highlight patient-reported events and the corresponding clinical actions, as well as lifestyle and PROs in participants who completed the intervention, with exploratory nutrigenomic and microbiota data.

### Baseline Participant Characteristics

Of the 20 patients with aLC enrolled, 14 completed the full 30-week intervention, which included attending all scheduled visits (V1-V10) and consistent submission of self-reported data through the app. Four patients did not complete the study due to death and 2 due to clinical and emotional deterioration. Baseline characteristics for all initially enrolled participants are summarized in Table 2; participants who discontinued the study were censored for longitudinal analyses.

**Table 2.** Baseline characteristics of participants enrolled in the ALIBIRD prospective pilot intervention for advanced lung cancer (N=20)^a^.

Characteristic	Values
Age (years), mean (SD)	
43-77	61.05 (9.70)
Sex, n (%)	
Female	12 (60)
Male	8 (40)
User technological level, n (%)	
Low (caregiver support)	6 (30)
Medium	9 (45)
High	5 (25)
Tobacco habit, n (%)	
Former smoker	10 (50)
No (never)	9 (45)
Yes	1 (5)
Tumor location, n (%)	
Lung	17 (85)
Pleura	1 (5)
Thymus	2 (10)
Stage of cancer, n (%)	
IIIB/C	2 (10)
IV	18 (90)
Eastern Cooperative Oncology Group^b^, n (%)	
0	11 (55)
1	8 (40)
2	1 (5)
Systemic treatment, n (%)	
CT^c^	3 (15)
IT^d^	1 (5)
CT-IT	7 (37)
TKI^e^	8 (40)
Radical therapy, n (%)	
CT-RT^f^	1 (5)

a Data include sociodemographic, clinical, and treatment-related variables. Continuous variables are expressed as mean (SD) and categorical variables as n (%).

b A performance status scale used in oncology.

c CT: chemotherapy.

d IT: immunotherapy.

e TKI: tyrosine kinase inhibitors.

f RT: radiotherapy.

### Usage Metrics, Data Completeness, and Usability Assessment

This section presents a comprehensive analysis of system usage, data collection patterns, and perceived usability, integrating insights from both patients and health care professionals. Table 3 summarizes the number of data entries provided by participants throughout the study period, offering insights into patient compliance and engagement levels.

**Table 3.** Summary of patient-generated data entries and wearable-derived records during the ALIBIRD mHealth intervention (N=20). The table reports the total number of automatic wearable recordings (physical activity and sleep), on-demand symptom and weight entries, and scheduled questionnaire submissions collected across all participants.

Data entries	Total records, n
Automated records^a^	
Physical activity	3589
Sleep	3468
On-demand manual records^b^	
Symptoms	679
Weight	123
Manual scheduled records^c^	
Dietary intake and appetite log	487
European Organisation for Research and Treatment of Cancer Quality of Life Questionnaire—Lung Cancer 13	170
Mediterranean Diet Adherence Screener	34
International Physical Activity Questionnaire short form	159
Pittsburgh Sleep Quality Index	53
Patient-Reported Outcomes version of the Common Terminology Criteria for Adverse Events	145
European Quality of Life-5 Dimensions 5 Levels	169
Hospital Anxiety and Depression Scale	51

a Automated records: entries automatically captured by wearable devices, such as physical activity and sleep data.

b On-demand manual records: entries made by patients whenever they experience symptoms or wish to log specific data, such as weight.

c Scheduled records: entries that patients complete at predefined intervals according to the study schedule, typically in the form of questionnaires.

**Figure 4.**
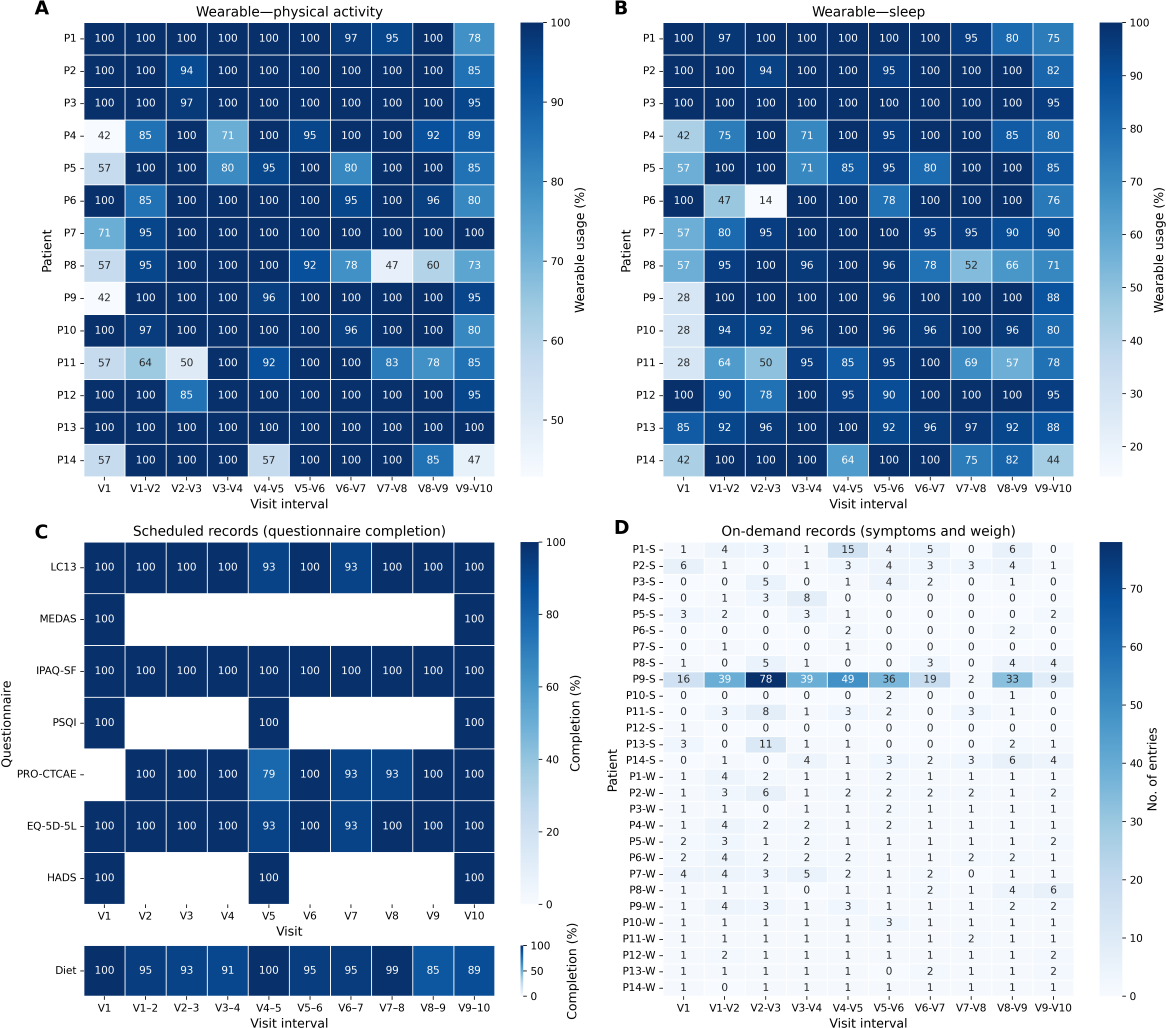
Individual-level data collection across visits by data type and recording modality (N=14). (**A**) Wearable—physical activity, (**B**) wearable—sleep, (**C**) scheduled records (questionnaire completion), and (**D**) on-demand records (symptoms and weight). Manual scheduled records include questionnaires with different expected schedules: QLQ-LC13, MEDAS, IPAQ-SF, and EQ-5D-5L were expected at each visit (V1-V10); PSQI and HADS were scheduled only at visits V1, V5, and V10; and PRO-CTCAE was expected from visit 2 to 10 (V2-V10). The dietary log was completed on a weekly basis, and adherence is represented using the intervals between visits. Percentages of adherence for dietary log and wearable monitoring are expressed relative to each patient’s expected entries, as visit intervals and monitoring periods varied across participants. EQ-5D-5L: European Quality of Life-5 Dimensions 5 Levels; HADS: Hospital Anxiety and Depression Scale; IPAQ-SF: International Physical Activity Questionnaire short form; LC13: Lung Cancer 13; MEDAS: Mediterranean Diet Adherence Screener; PRO-CTCAE: Patient-Reported Outcomes version of the Common Terminology Criteria for Adverse Events; PSQI: Pittsburgh Sleep Quality Index.

Across the 14 participants who completed the intervention, adherence to scheduled questionnaires remained high throughout the 30-week follow-up. Completion rates ranged from 94% for dietary records to 100% for several instruments, with visit-level averages between 95% and 100%. Wearable-based passive monitoring showed adherence between 66% and 96% across visits, with sleep tracking representing the lower range and physical activity data showing slightly higher and more stable capture. Symptom reporting was heterogeneous, with peaks during the early-mid follow-up and variable contributions among patients, while weight entries were more evenly distributed, with at least 1 measurement recorded by most participants between consecutive visits. Figure 4 illustrates individual-level data collection across visits, including wearable-derived physical activity (Figure 4A), wearable-derived sleep data (Figure 4B), scheduled questionnaire completion (Figure 4C), and on-demand records for symptoms and weight (Figure 4D). This visualization highlights variations in engagement over time and among participants, helping to identify adherence patterns and potential gaps in data reporting.

In addition to evaluating what data were collected, it is also important to understand how patients interacted with the application interface. App usage data revealed that, on average, participants spent the largest proportion of their app interactions on the home screen (33%), followed by the weekly tasks (19%—which include actions related to diet and other lifestyle variables such as sleep, mood, energy, and gastrointestinal rhythm), symptom reporting (15%), and the notifications or message box (10%—which include automated notifications and clinic-patient exchanges). These percentages represent the mean proportion of interactions per patient, calculated relative to each individual’s total app interactions and then averaged across participants.

**Figure 5.**
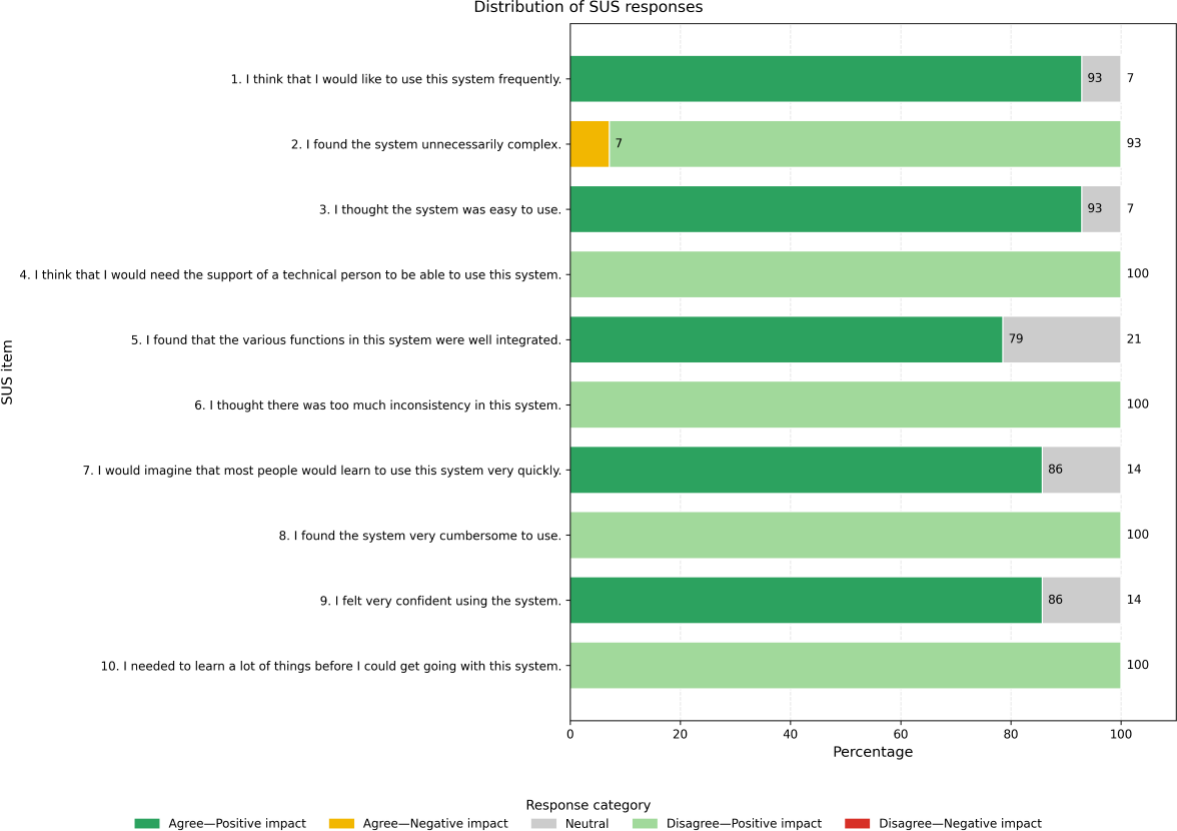
Distribution of System Usability Scale responses categorized by agreement and their impact on usability (N=14). The bar chart shows the distribution of responses for each of the 10 System Usability Scale items. Items 1, 3, 5, 7, and 9 are positively phrased, where Agree/Strongly agree responses indicate positive usability and Disagree/Strongly disagree responses indicate negative usability. Items 2, 4, 6, 8, and 10 are negatively phrased, where Disagree/Strongly disagree responses indicate positive usability and Agree/Strongly agree responses indicate negative usability. Neutral responses correspond to the midpoint of the Likert scale (Neither agree nor disagree). The “Disagree—Negative Impact” category is included for completeness, although no responses were observed in this category. SUS: System Usability Scale.

The average SUS score among patients who completed the study (n=14) was 90, indicating a high level of usability for the mobile app. This value is well above the commonly accepted benchmark of 68, which is considered the threshold for above-average systems. Figure 5 displays the distribution of responses for each item of the SUS questionnaire, offering a visual overview of user sentiment across all items. The consistently positive responses suggest a strong perception of ease of use and user satisfaction among participants.

PREMs provided valuable insights into the participants’ perception of the ALIBIRD platform. Regarding the question “So far, the app has motivated me to follow guidelines to better control possible symptoms and adopt healthy lifestyle recommendations,” the majority of participants responded positively, with 9 out of 14 (64%) indicating “Strongly agree” and 3 out of 14 (21%) indicating “Agree,” while 2 out of 14 (14%) remained neutral. For the question “How would you rate your level of satisfaction with the app and the follow-up you receive in the study?,” 50% of the participants reported being “Completely satisfied” and the other 7 out of 14 (50%) being “Satisfied.” Finally, in response to “Would you recommend an app with similar features and follow-up to someone you know who is in your situation?,” all participants (14/14, 100%) responded “Yes.”

In addition, usability was also assessed among health care professionals using the clinical web-based interface. The mean SUS score among professionals (n=5) was 86, reflecting a positive perception of the platform’s usability in clinical practice. The evaluation involved 5 members of the clinical team, including oncologists, a nutritionist, and a nurse.

### Evaluation of Outcome Data

In this section, we analyze the PRO and PROM results to assess the overall clinical impact of ALIBIRD mHealth platform, focusing on both clinically actionable events and patient-reported measures collected during follow-up. Outcomes include the detection and management of symptom- and nutrition-related alerts that prompted clinical actions, as well as longitudinal changes in lifestyle behaviors, symptoms, and quality-of-life indicators reported by patients. Together, these results provide an integrated view of the platform’s clinical impact and its role in supporting patient monitoring and care during the intervention.

#### Clinical Actions Triggered by Reported Events

Clinical actions were driven by events captured and processed via the ALIBIRD mHealth platform, including patient-reported symptoms and nutritional indicators, which triggered automated alerts and subsequent interventions by the health care team. During the study, the most frequently reported symptoms among patients (n=20) were asthenia (164/679, 24.2%) and dyspnea (132/679, 19.4%). Overall, most symptom reports were classified as mild (grade 1: 367/679, 54.0%), followed by moderate (grade 2: 238/679, 35.1%) and severe (grade 3: 74/679, 10.9%).

These patient-reported symptoms, combined with nutritional monitoring, triggered the majority of the 2100 automated recommendations and 143 clinical alerts generated by the platform for the health care team. Symptom-related alerts (75/143, 52.4%) were most often due to constipation (28/75, 37%), pain (19/75, 25%), and dyspnea (9/75, 12%), followed by fever (9/75, 12%) and less frequent events such as hemoptysis (4/75, 5%), diarrhea, drowsiness, and vomiting, each (2/75, 3%). Nutritional alerts represented 47.6% (68/143), mainly related to reduced food intake (52/68, 76%), fewer than 3 meals per day (9/68, 13%), and weight loss greater than 5% (7/68, 10%).

Health care professionals managed these alerts using the ALIBIRD web-based tool to resolve alerts, which led to 42 phone calls and 20 in-app messages to patients. The mean average resolution time for an alert was 2.05 (1.72 SD) days. Clinical responses included 52 therapeutic adjustments, 10 expedited medical visits, 15 referrals to other specialties, 2 emergency referrals, and 4 imaging reevaluations, with 2 cases confirming clinical-radiological progression.

#### Lifestyle and PROs

Among patients who completed the intervention, several lifestyle and patient-reported outcome measures were monitored over time. Adherence to the Mediterranean diet (Mediterranean Diet Adherence Screener) increased from 50% (7/14) at baseline (V1) to 71% (10/14) by the end of the intervention (V10), while BMI remained relatively stable across all visits, with no clinically meaningful changes.

**Figure 6.**
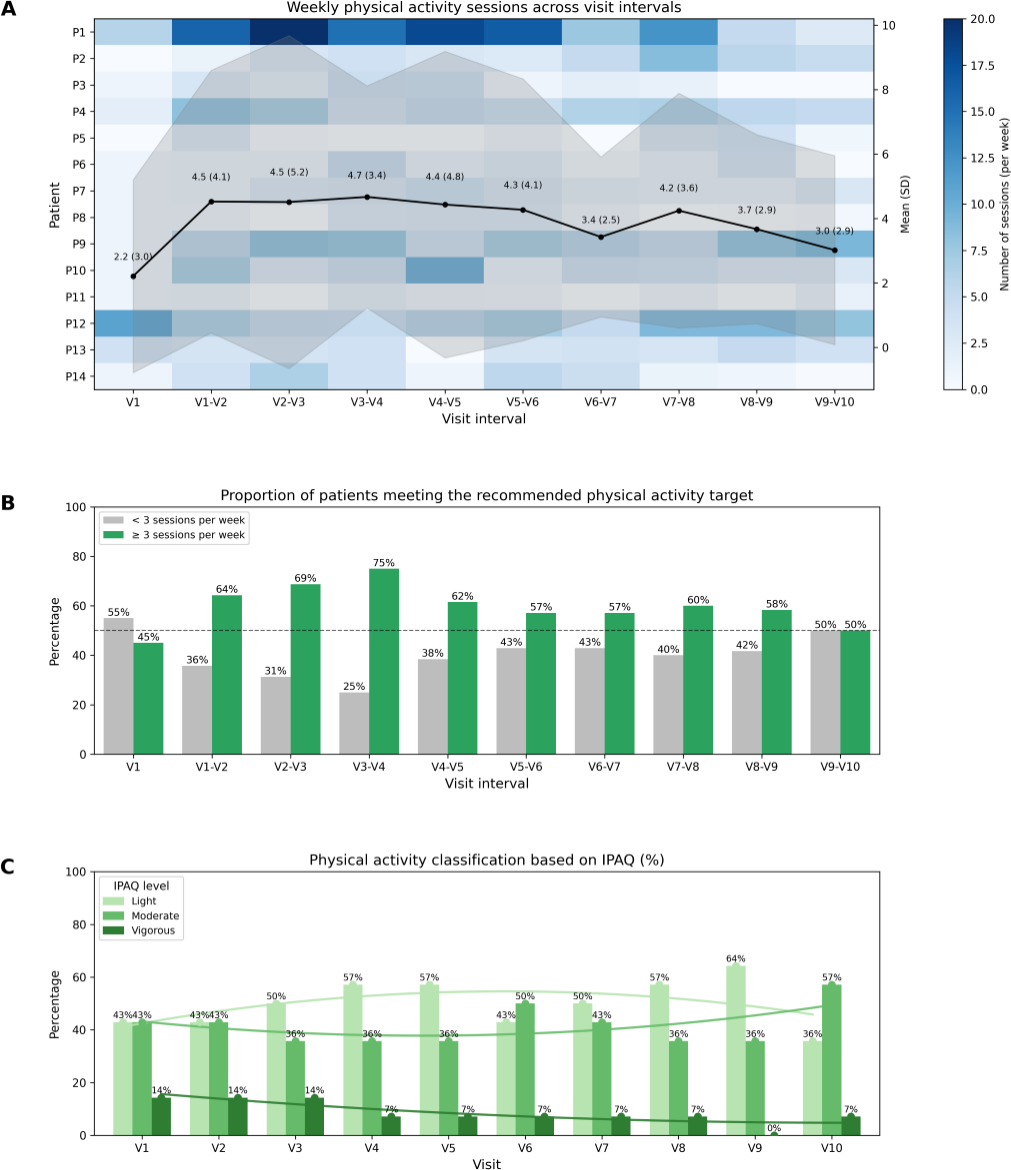
Physical activity outcomes across the study period (N=14). (**A**) Heatmap showing the average number of weekly physical activity sessions recorded via Fitbit across visit intervals, with overlaid mean (SD). (**B**) Percentage of patients who met the recommended physical activity target of ≥3 sessions per week compared with those who did not, aggregated by visit interval. (**C**) Distribution of physical activity intensity levels (light, moderate, and vigorous) based on International Physical Activity Questionnaire short form responses across study visits. IPAQ: International Physical Activity Questionnaire.

Physical activity, monitored via the ALIBIRD mobile app and Fitbit devices, showed that participants performed a mean average of 3.90 (SD 3.68) sessions per week and 6087 daily steps (SD 2675). Figure 6 illustrates the evolution of these metrics across the study period, including the number of weekly physical activity sessions, adherence to the recommended target of 3 or more sessions per week, and the distribution of activity intensity levels (light, moderate, and vigorous) according to the International Physical Activity Questionnaire short form questionnaire. Figure 6 summarizes physical activity outcomes across the study period, including weekly exercise frequency (Figure 6A), the proportion of patients meeting the recommended target of ≥3 sessions per week (Figure 6B), and activity intensity levels based on the International Physical Activity Questionnaire short form results (Figure 6C). Step count data collected through Fitbit indicated predominantly light levels of physical activity, with relatively stable averages across visits.

Sleep monitoring revealed an average duration of 7.61 (SD 1.34) hours per day according to Fitbit data, consistent with recommended sleep duration. However, Pittsburgh Sleep Quality Index results indicated persistently poor subjective sleep quality among most participants. The proportion of patients classified as having poor sleep quality was 71.4% (10/14) at baseline, slightly improved at midintervention (V5) to 64.3% (9/14), and worsened again by the end of the study (V10) to 78.6% (11/14).

Regarding emotional health, Hospital Anxiety and Depression Scale—A indicated a slight increase in clinically relevant anxiety from 0% (0/14) to 7% (1/14), while Hospital Anxiety and Depression Scale—D revealed that 14% (2/14) of patients developed depressive symptoms by V10. In relation to patient QoL and specific symptoms, the European Organisation for Research and Treatment of Cancer Quality of Life Questionnaire—Lung Cancer 13 questionnaire generated a total of 655 symptom reports throughout the study. The most frequently reported symptoms were total pain (242/655, 36.9% of reports), cough (115/655, 17.6%), and total dyspnea (113/655, 17.3%). In addition, the Patient-Reported Outcomes version of the Common Terminology Criteria for Adverse Events questionnaire, which captured 5025 patient-reported toxicity items over the 10 study visits, highlighted the most frequent symptom groups as follows: sleep and wake patterns (962/5025, 19.1% of reports), pain (847/5025, 16.9%), and neurological and perception disorders (774/5025, 15.4%). QoL, assessed by the European Quality of Life-5 Dimensions 5 Levels questionnaire, remained stable throughout the study. The mean score at baseline was 0.86 (SD 0.10) and showed only minor variations across visits, with a slight increase at midintervention (V4: mean 0.92, SD 0.09) and a final mean score of 0.88 (SD 0.15) at V10. Overall, participants maintained good perceived health status during the intervention, with no clinically relevant decline or improvement observed. Table 4 provides a summary of the pre- and postintervention comparison between baseline (V1) and the end of the study (V10) for participants who completed the intervention.

**Table 4.** Pre- and postintervention comparison of dietary, nutritional, physical activity, and sleep parameters between baseline (V1) and end of study (V10) among participants who completed the 30-week ALIBIRD mHealth intervention (N=14). Statistical significance was assessed using paired tests or McNemar test where applicable^a^.

Variables	Preintervention	Postintervention	*P* value
	V1	V10	
Diet			
Mediterranean Diet Adherence Screener, % of adherence (n)	50 (7/14)	71 (10/14)	.12
Food intake reduction, percentage of patients (n)			
No	43 (6/14)	43 (6/14)	—^b^
Mild-moderate	57 (8/14)	50 (7/14)	—^b^
Severe	0 (0/14)	7 (1/14)	—^b^
Dietary guideline compliance,^c^ % of patients, (n)			
Meat, eggs, fish, legumes, and dairy	21 (3/14)	57 (8/14)	.06
Fruits and vegetables	0 (0/14)	57 (8/14)	—^b^
Liquids	0 (0/14)	100 (14/14)	—^b^
Cold meat	43 (6/14)	71 (10/14)	.22
Pastries	21 (3/14)	50 (7/14)	.13
Sugary drinks and sweets	79 (11/14)	71 (10/14)	1
Alcohol	64 (9/14)	71 (10/14)	1
Nutritional status			
Global Leadership Initiative on Malnutrition Criteria, % of patients (n)			
Normonutrition	79 (11/14)	79 (11/14)	.26
Moderate malnutrition	14 (2/14)	36 (5/14)	
Severe malnutrition	7 (1/14)	14 (2/14)	
BMI, mean (SD)	26.14 (2.93)	26.13 (3.08)	.99
Muscle ultrasonography, mean (SD)			
Area, cm²	4.43 (1.40)	4.50 (0.91)	.80
y-axis in relaxation, cm	1.41 (0.39)	1.43 (0.21)	.81
y-axis in contraction, cm	1.57 (0.48)	1.52 (0.42)	.77
x-axis, cm	3.92 (0.72)	3.76 (0.50)	.44
Bioimpedance (ohms), mean (SD)	526.93 (7.68)	508.86 (79.9)	.42
Physical activity, mean (SD)			
Weekly sessions	2.21 (2.88)	3.02 (2.82)	.34
Daily steps	3945 (2015)	4975 (2409)	.19
International Physical Activity Questionnaire short form, % of patients (n)			
Walking	43 (6/14)	36 (5/14)	.53
Moderate	43 (6/14)	57 (8/14)	
Vigorous	14 (2/14)	7 (1/14)	
Sleep			
Duration (hours)	6.93 (1.62)	7.07 (1.18)	.75
Pittsburgh Sleep Quality Index, % of patients (n)			
Poor quality	71 (10/14)	79 (11/14)	.53
Good quality	29 (4/14)	21 (3/14)	

a Percentages refer to patients; absolute numbers are indicated within parentheses (n/N).

b The *P* value could not be calculated. In some analyses, the McNemar test could not be performed due to the lack of variability in the data. Specifically, when one of the categorical variables involved is constant, meaning all responses fall within a single category, statistical tests that rely on variation between groups, such as McNemar test, are unable to calculate an associated *P* value or significance measure.

c Dietary recommendations: meat + eggs: minimum 4-6 servings per week; fish + shellfish: minimum 3-4 servings per week; legumes: minimum 2-4 servings per week; dairy products: minimum 2 servings per day; fruit + vegetables: minimum 5 servings per day; liquids (water, sugar-free isotonic drinks, infusions, coffee, juices, etc): minimum 4 servings (glasses) or 1 liter per day; processed meats: maximum 1-2 servings per week; pastries: maximum 1-2 servings per week; sugary drinks and sweets: maximum 1-2 servings per week; and alcohol: do not consume.

### Exploratory Nutrigenetic and Gut Microbiota Profiling

As components of the personalized intervention, baseline nutrigenetic analysis and gut microbiota profiling were incorporated into the platform to inform individualized nutritional recommendations. In this study, these measures were not evaluated as clinical outcomes; rather, descriptive summaries of the baseline profiles (and V10 profiles for microbiota) are presented to contextualize patient heterogeneity.

Genetic analysis was performed on all initial participants (n=20), targeting 20 variants associated with lifestyle habits, nutrition, and cancer. The analysis revealed that 60% of the population carried a CLOCK gene variant associated with protection against sleepiness, favorable breakfast timing, and enhanced intestinal motility, which may support appetite and dietary intake during cancer treatment. Most participants did not present metabolic alterations for gluten sensitivity (16/20, 80%) or lactose intolerance (15/20, 75%), although 5 patients exhibited lack of lactase persistence and were recommended a lactose-free diet. Only 25% (5/20) of the sample displayed a favorable genetic profile for systemic inflammation, which may contribute to better clinical outcomes and reduce circulating proinflammatory factors. Detailed results of the nutrigenetic analysis are provided in Table S1 in Multimedia Appendix 4.

Gut microbiota analyses were conducted in the subset of participants who completed the intervention and had valid samples available at both baseline and end of intervention (n=13). At baseline (V1), 38.5% (5/13) of the participants exhibited a gut microbiota profile similar to the healthy population (dysbiosis grade <30%), whereas 61.5% (8/13) showed a clear state of dysbiosis (dysbiosis grade >30%). After completing the intervention (V10), 15.4% (2/13) of the participants showed improvement in dysbiosis grade, 15.3% (2/13) maintained their baseline status, 23.1% (3/13) experienced a slight deterioration, and 46.2% (6/13) presented a marked deterioration.

## Discussion

### Principal Findings

This pilot study demonstrates the feasibility, usability, and acceptability of the ALIBIRD mHealth platform as a digital health solution to support the care of patients with aLC. The ALIBIRD apps achieved a mean SUS score of 90 of 100 among patients and 86 of 100 among health care professionals, reflecting excellent usability across both user groups. These results were complemented by the PREMs, which showed that 100% of the participants were satisfied and willing to recommend the platform, and 85% of the participants expressed high motivation to follow the recommendations received. Together with the high completion rates of scheduled questionnaires (94%‐100%) and sustained wearable adherence (81%‐96% after the initial week), these findings indicate strong engagement and adherence over the 30-week follow-up.

Patients actively used the mobile app and wearable device to report symptoms, record lifestyle data, and track physical activity and sleep, while clinicians remotely reviewed this information to guide interventions. A total of 679 symptoms and 143 clinical alerts were processed, with a mean resolution time of 2.05 days. Notably, 2 alerts led to early identification of unsuspected disease progression, underscoring the potential value of continuous digital monitoring in supporting timely clinical decision-making. Importantly, lifestyle-related indicators showed favorable trends during the intervention: adherence to the Mediterranean diet increased from 50% to 71%, physical activity levels improved modestly, and sleep duration averaged 7.6 hours per night. Although subjective sleep quality (Pittsburgh Sleep Quality Index) remained poor for most participants, these findings highlight the potential of the platform to promote awareness and gradual behavioral changes in diet and activity.

QoL, as measured by European Quality of Life-5 Dimensions 5 Levels, remained stable throughout the study, suggesting that continuous monitoring and early interventions may help preserve well-being in this patient population. This stability is particularly relevant, given the typically unfavorable clinical course of aLC. Taken together, these results confirm the feasibility and usability of ALIBIRD for sustained multimodal engagement in long-term digital oncology interventions.

### Comparison With Prior Work

The introduction of PROMs into routine clinical practice has become increasingly important in cancer care, as these tools provide a structured way to assess patients’ health status and monitor disease evolution. However, their routine implementation still faces practical barriers, including workload burden, the need for training and support, associated costs, and reluctance among some clinicians. The digital collection of PROMs (ePROMs) has emerged as a strategy to overcome many of these limitations by enabling more efficient patient monitoring and supporting timely clinical decision-making. Evidence from previous trials also indicates that ePROM-based follow-up can contribute to improved clinical outcomes and has the potential to optimize health care resource use [10,11]. In this context, digital health platforms such as ALIBIRD can help overcome routine implementation barriers by facilitating automated data collection and generating clinically actionable information.

The usability, adherence, and engagement results observed in this study compare favorably with previous long-term mHealth interventions in oncology and chronic disease management [25,64]. Anders et al [24] described SUS scores of 85‐89 in patients with breast cancer, Kessel et al [26] reported 83% willingness to continue app use, and Wu et al [25] documented 61%‐77% daily engagement in long-term self-management apps. ALIBIRD’s higher usability scores and sustained adherence likely reflect its integrative design, combining active self-reporting, passive monitoring, personalized recommendations, and timely clinician-patient communication.

In addition, the high levels of satisfaction and motivation reflected by the PREMs align with prior findings that emphasize the role of positive user experience in sustaining engagement and promoting self-care behaviors in digital health interventions [65,66]. Our results also support evidence that digital health tools can enhance self-management, enable early symptom detection, and optimize patient-centered care [25,28].

In this study, frequent alerts related to constipation, pain, dyspnea, and nutritional decline were effectively addressed through remote interactions, demonstrating how digital platforms can extend clinical follow-up beyond hospital visits. The improvements in dietary adherence and weight stabilization observed among participants are particularly relevant, as they suggest that app-based guidance can mitigate the nutritional challenges and risk of malnutrition typically observed in patients with aLC [67-69]. This may help explain the stable quality-of-life scores observed in our cohort, contrasting with the decline usually associated with disease progression.

A distinctive aspect of ALIBIRD is its integration of precision-nutrition elements, including nutrigenetic and microbiota analyses. This approach aligns with emerging evidence on the role of gut health and genetic predispositions in modulating treatment response and nutritional outcomes in cancer [70-77]. The prevalence of dysbiosis and inflammation-related variants in this cohort reinforces the importance of combining molecular data with behavioral and lifestyle tracking to support personalized, adaptive interventions.

### Strengths and Limitations

Implementing PRO-based monitoring between clinic visits offers clear advantages in terms of accessibility and closer patient follow-up. At the same time, it also introduces several operational challenges, such as ensuring reliable and robust platform performance, maintaining completion adherence, and providing clinicians with timely information to support clinical decision-making. Within this context, this study provides early evidence supporting the feasibility of deploying an integrated, multidomain digital health platform in oncology care for patients with aLC. The combined monitoring and response to clinical symptoms, lifestyle behaviors, and personalized nutrition represent a holistic approach rarely evaluated in real-world settings. Another strength is the dual usability assessment among patients and health care professionals, which offers insight into cross-stakeholder acceptance—an essential component for successful digital implementation in clinical workflows.

However, several limitations should be acknowledged. The small sample size (n=20) and the single-arm design limit statistical power and external generalizability. Of the 20 participants initially enrolled, 6 discontinued early—4 due to rapid clinical deterioration related to disease progression and 2 due to worsening emotional well-being—illustrating the clinical fragility of this population rather than limitations of the digital health solution itself. Importantly, despite the high clinical vulnerability commonly observed in aLC, most patients who remained in the study were able to sustain meaningful engagement with the platform. This may have been facilitated by the frequency of in-person visits, family or caregiver support, and the involvement of both the clinical team and the biomedical engineering support. These combined elements may have helped reinforce motivation and support sustained engagement with the digital health solution.

Other populations with less frequent clinical contact or fewer support resources may require additional strategies—such as adaptive reminders, motivational feedback, or gamification—to maintain long-term engagement. In addition, the economic implications of implementing such digital health interventions were not assessed in this study, and future research should examine cost-effectiveness and scalability in routine practice.

Finally, while this study confirmed feasibility, usability, and high user satisfaction, several findings suggest the potential for meaningful clinical benefits. The rapid resolution of a substantial number of clinical alerts, the remote identification of unsuspected tumor progression in 2 patients, and the preservation of QoL and healthy lifestyle behaviors over time indicate that the intervention may have supported early clinical decision-making and proactive patient management. Nonetheless, as a pilot study with a limited sample size, it was not powered to evaluate clinical outcomes or survival effects, which should be investigated in larger controlled trials.

### Conclusions

Future studies should aim to validate these findings in larger, multicenter randomized trials and assess the impact of the ALIBIRD mHealth platform on clinical outcomes, nutritional status, and QoL over longer periods. Integration of artificial intelligence models could further enhance the platform’s predictive capacity, enabling personalized alerts, adaptive feedback, and early detection of adverse events.

From a clinical perspective, ALIBIRD demonstrates the potential of combining digital monitoring, precision nutrition, and PROs to enable more proactive and individualized oncology care. Its holistic, multidisciplinary design supports continuous data collection and facilitates the incorporation of lifestyle, molecular, and behavioral variables into personalized interventions.

Moving forward, early-stage work is currently focused on 2 key areas. The first involves adapting the platform for use in other tumor types and in clinical scenarios with lower symptom burden, such as survivorship care or follow-up of patients in remission, enabling customization to both tumor-specific and individual patient needs. The second area relates to the design of a new care model within the oncology department to manage the continuous flow of patient-generated information and support earlier clinical responses. This will require establishing coordinated workflows involving health care professionals and biomedical engineering teams to ensure efficient data management and timely intervention.

In summary, this pilot study highlights the high usability, sustained adherence, and positive patient experience achieved with the ALIBIRD mHealth platform. These results underscore its feasibility for integration into routine oncology care and lay the groundwork for future research aimed at scaling up digital health, patient-centered strategies in cancer management.

## Supplementary material

10.2196/69525 Multimedia Appendix 1 Technical description and development of the ALIBIRD mHealth platform.

10.2196/69525 Multimedia Appendix 2 Rule engine and patient-monitoring dimensions of the ALIBIRD platform.

10.2196/69525 Multimedia Appendix 3 Full methods for nutrigenetics and gut microbiota.

10.2196/69525 Multimedia Appendix 4 Detailed nutrigenetic results.

## Abbreviations

aLC: advanced lung cancer
LC: lung cancer
MEDAS: Mediterranean Diet Adherence Screener
mHealth: mobile health
NSCLC: non–small cell lung cancer
PREM: patient-reported experience measure
PRO: patient-reported outcome
PROM: patient-reported outcome measure
QoL: quality of life
REDCap: Research Electronic Data Capture
SNP: single nucleotide polymorphism
SUS: System Usability Scale
