# Supplementary material for: Evaluation of the ALIBIRD mHealth Platform for Care of Patients With Lung Cancer: Prospective Pilot Study
Source: JMIR Cancer. 2026 Feb 11;12:e69525. doi: 10.2196/69525 (PMC12893646; doi:10.2196/69525)
Supplement: Multimedia Appendix 1 [file cancer-v12-e69525-s001.pdf]

Multimedia Appendix 1: Technical Description and Development of the ALIBIRD mHealth Platform. This supplementary material provides additional technical details on the development, design, and functionalities of the ALIBIRD mHealth platform. It includes an overview of the participatory and agile methodologies applied during the creation process, as well as screenshots of the mobile app for patients and the web-based dashboard for healthcare professionals. These details complement the brief description provided in the main manuscript and aim to offer deeper insights into the platform's architecture and user interface design. *Figures and tables are numbered starting from S1 within each Multimedia Appendix.*

Participatory design, as described in the literature, involves engaging end users in all phases of design, development, and implementation to ensure that mHealth technologies are useful, acceptable, and appropriately tailored to the clinical context. This approach facilitated the identification of the needs of both patients and healthcare professionals, as well as the key functionalities required for the applications. Through surveys, interviews, and focus groups with patients, healthcare professionals, and researchers, we assessed the clinical context and identified factors that facilitate cancer prognoses via telemonitoring and digital health. The results of these analyses allowed us to define the main design priorities and outline the core system characteristics (Textbox S1).

Textbox S1. Activities during the ideation and design stages of the ALIBIRD platform.

1. Surveys and individual interviews. Information collected included: sociodemographic characteristics, history with the disease, perceived quality of service and care, aspects related to medication control, management of medical appointments, previous experience with the use of technology and smart devices, and the availability of family and/or caregiver support.
2. Focus groups. A total of 4 patients, 5 health professionals (2 oncologists, 2 nutritionists, and 1 nurse), and 3 biomedical engineers participated. Based on the patients' daily life routines and the impact of the disease, we identified patient needs and desired functionalities for a mobile application aimed at self-management of health and telemonitoring of cancer patients. This resulted in the design of a mockup application including its main graphical user interfaces needed to collect and communicate the necessary variables and PROs of interest.
3. Prototype testing. Prototypes of the mobile app for patients and of the web application for professionals were developed and continuously tested, collecting feedback from both patients and professionals to ensure that all data and communication requirements were met.

The platform development followed an Agile methodology, an iterative and incremental approach that emphasizes flexibility and continuous improvement. The process included several co-creation phases, such as analysis and ideation, design and development, and requirement specification. To design the system logic and components, we considered the clinical dimensions and variables relevant to cancer patients, data sources, and instruments for data capture. The primary function of the ALIBIRD platform is to provide essential information for cancer patient care and monitoring through a mobile app for patients and a

web tool for healthcare professionals, both supported by backend infrastructure and cloud-based services.

The mobile app and the clinical web-based tool were developed using cross-platform hybrid technologies, specifically the Ionic Framework with TypeScript, HTML, CSS, and Angular. The consistent use of these technologies reduces development complexity and lowers maintenance costs. The backend was built with NestJS and NodeJS frameworks, integrating a JSON Rules Engine to automatically process patient-reported outcomes (PROs) and generate alerts and personalized recommendations. Data storage is managed by a MongoDB database server (Community version). Backend services also include Firebase for authentication, REDCap as an electronic case report form (eCRF) tool, the Fitbit web API for collecting wearable data, and OneSignal for push notification.

The mobile app allows patients to report PROs and PROMs related to symptoms and lifestyle. It can also synchronize with Fitbit devices to display physical activity and sleep data. The backend processes all captured information and provides personalized recommendations, some based on nutrigenetics. Additionally, the app delivers weekly progress summaries, results of nutrigenetic and gut microbiota analyses, and educational materials aimed at supporting patient self-management (Figure S1).

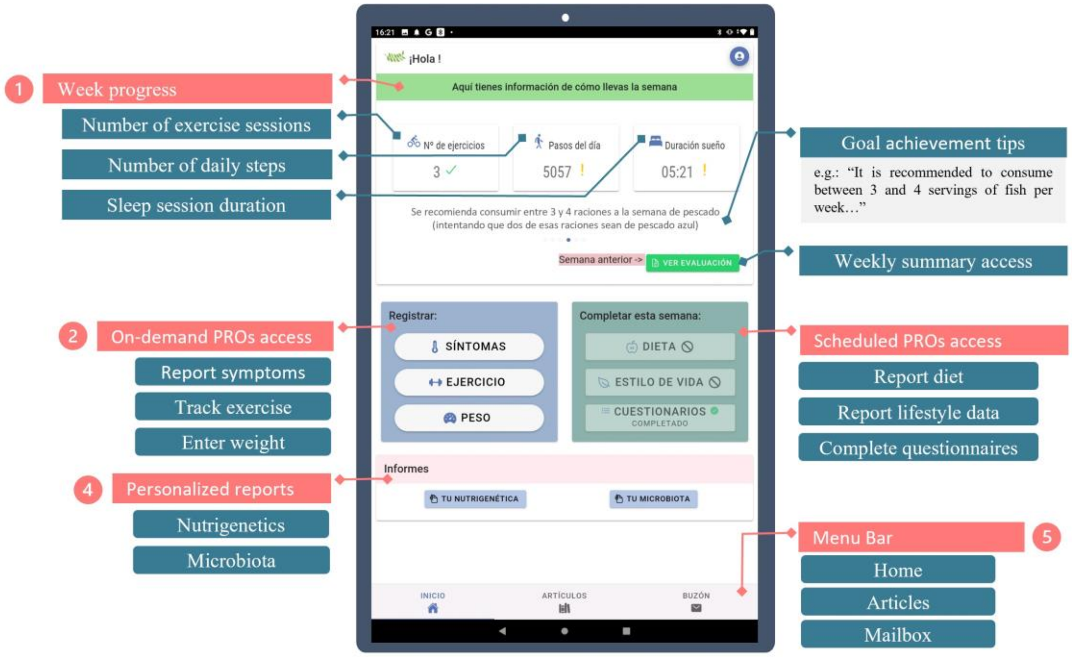

Figure S1. Main screen of the ALIBIRD mobile app (Spanish version), featuring: (1) Weekly Progress, which displays exercise sessions, daily steps, sleep duration, recent activity tips, and access to a detailed weekly summary; (2) On-demand PROs, enabling users to report symptoms, track physical activity, and log weight for BMI monitoring; (3) Scheduled PROs, for weekly diet logging, lifestyle questions, and validated questionnaires; (4) Personalized

reports, providing access to nutrigenetic and microbiota analyses with tailored recommendations; (5) Menu bar, offering links to the main screen, educational resources, and messages/notifications.

The system automatically processes all patient information and makes it available to healthcare professionals through a web-based dashboard, allowing remote monitoring of symptoms, nutritional status, and lifestyle patterns (Figure S2).

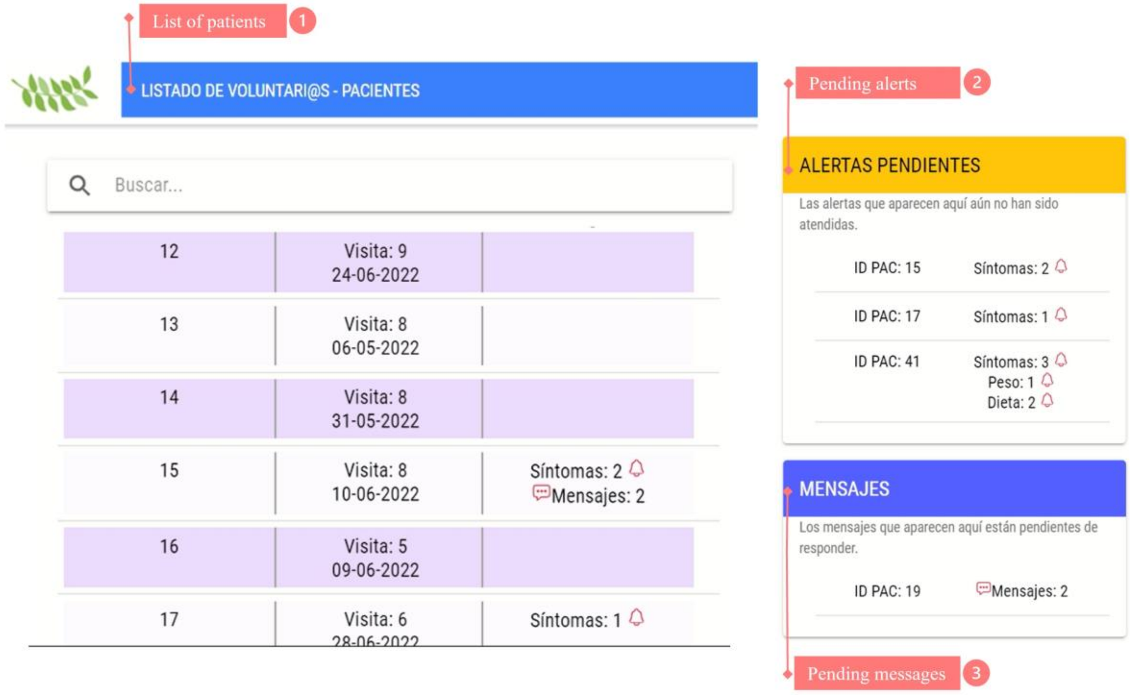

Figure S2. Screenshots of the web-based tool for healthcare professionals (Spanish version): (1) Section with a table listing patients (column 1: patient ID, column 2: number and date of the last medical visit, column 3: number of pending alerts and messages); (2) Section with a table of pending alerts (column 1: patient ID, column 2: type and number of alerts); (3) Section with a table of pending messages (column 1: patient ID, column 2: number of pending messages). All components are interactive, allowing healthcare professionals to click on a patient, alert, or message to access specific information.
