# Supplementary material for: Evaluation of the ALIBIRD mHealth Platform for Care of Patients With Lung Cancer: Prospective Pilot Study
Source: JMIR Cancer. 2026 Feb 11;12:e69525. doi: 10.2196/69525 (PMC12893646; doi:10.2196/69525)
Supplement: Multimedia Appendix 2 [file cancer-v12-e69525-s002.pdf]

Multimedia Appendix 2: Rule Engine and Patient Monitoring Dimensions of the ALIBIRD Platform. This supplementary material provides a detailed description of the rule-based engine implemented in the ALIBIRD mHealth platform, which is responsible for generating personalized recommendations and clinical alerts. *Figures and tables are numbered starting from S1 within each Multimedia Appendix.*

The engine was iteratively designed and refined during the software development process, incorporating expert feedback primarily from oncologists, nutritionists, and nursing professionals, and tested through simulated clinical scenarios to verify the accuracy and relevance of the outputs. This process ensured that the decision rules, thresholds, and outputs were clinically meaningful and aligned with established clinical guidelines and practice. The outputs generated by the ALIBIRD rule engine are grouped into four categories, each serving a specific purpose in supporting patient self-management and clinical decision-making:

- **Reminders:** Support patient adherence by prompting daily or weekly data entry and completion of scheduled questionnaires.
- **Positive Reinforcement:** Provide information and encourage goal achievement to promote healthy habits.
- **Weekly Summary:** Deliver weekly reports on progress across monitored variables and reinforce healthy behaviors.
- **Alerts:** Support patient monitoring by notifying healthcare professionals of critical events or changes requiring clinical attention.

Table S1 summarizes the main dimensions monitored by the platform and the patient-reported outcomes (PROs) used for each, along with the corresponding feedback generated by the rule engine.

Table S1. Dimensions monitored by the ALIBIRD platform, associated patient-reported outcomes (PROs), and types of feedback generated by the rule engine.

Dimension	PROs Evaluated	Type of Feedback
Diet and Nutrition	Number of meals, appetite level, intake reduction, portions of food groups, alcohol/tobacco consumption	Reminders, Positive Reinforcement, Weekly Summaries, Alerts
Physical Activity	Number of sessions, activity intensity	Reminders, Positive Reinforcement, Weekly Summaries
Sleep and Rest	Sleep duration, perceived rest quality	Reminders, Positive Reinforcement, Weekly Summaries
Symptoms	Symptom type and severity	Reminders, Positive Reinforcement, Alerts
Body Weight	% change in weight, Body Mass Index (BMI)	Alerts
Other Lifestyle Factors	Tobacco use, gastrointestinal rhythm, mood, energy level	Reminders, Positive Reinforcement, Weekly Summaries

Figure S1 provides a schematic overview of how patient data are evaluated, processed, and transformed into actionable outputs for both patients and healthcare professionals.

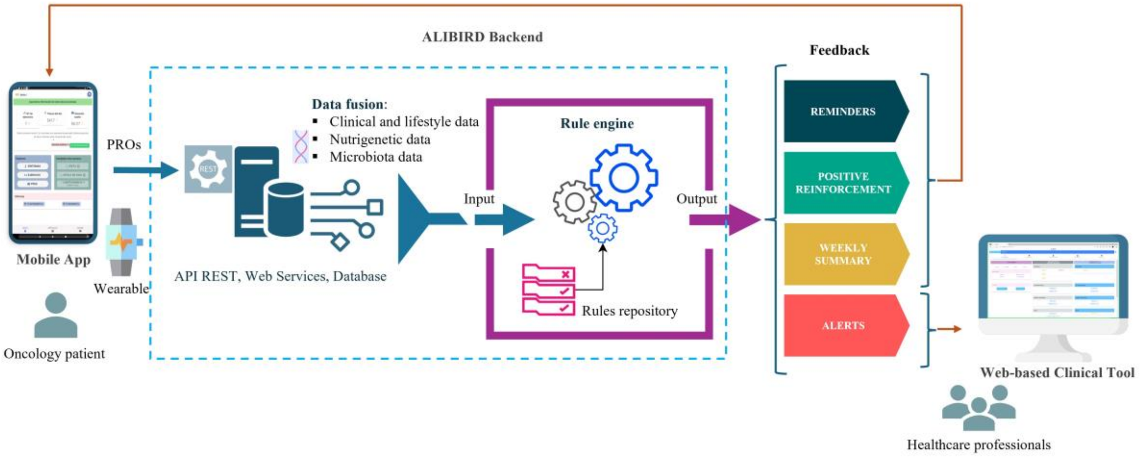

Figure S1. Schematic representation of the ALIBIRD platform's rule engine, illustrating how patient data are evaluated and transformed into reminders, weekly summaries, and clinical alerts for healthcare professionals.
