# Supplementary material for: Evaluation of the ALIBIRD mHealth Platform for Care of Patients With Lung Cancer: Prospective Pilot Study
Source: JMIR Cancer. 2026 Feb 11;12:e69525. doi: 10.2196/69525 (PMC12893646; doi:10.2196/69525)
Supplement: Multimedia Appendix 3 [file cancer-v12-e69525-s003.pdf]

Multimedia Appendix 3: **Full Methods for Nutrigenetics and Gut Microbiota.** *Figures and tables are numbered starting from S1 within each Multimedia Appendix.*

Nutrigenetics

Saliva samples were collected during the initial visit (V1) to identify genes involved in metabolism, nutrition, predispositions to sleepiness or constipation/diarrhea, gluten tolerance, lactose metabolism, appetite level, caffeine metabolism, and related traits. Genomic DNA was extracted with a Speedtools Tissue DNA extraction kit (Biotools B&M Labs. Inc., Spain) following the manufacturer's instructions [1]. Genotyping was performed using TaqMan Assays on a QuantStudio Real-Time PCR system (Thermo Fisher Scientific Inc., USA) according to the manufacturer's instructions [2].

Results were based on single nucleotide polymorphisms (SNPs) and composite scores derived from multiple SNPs. Each SNP was assigned a weight according to the strength of scientific evidence (high = 1, medium = 0.5, low = 0.25). Favorable, intermediate, or unfavorable genotypes contributed to an overall score that guided the personalized nutritional recommendations provided to patients.

Gut Microbiota

Fecal samples were collected at baseline (V1) and at the end of the intervention (V10) to evaluate the dysbiosis status of the participants and its evolution during the study. Total DNA was extracted using an optimized protocol based on the QIAamp DNA Stool Mini Kit (Qiagen Ltd., Germany). The V3-V4 region of the 16S rRNA gene was amplified following standard protocols [3]. Taxonomic assignment was performed using the naïve Bayesian classifier in DADA2, with Silva v.138 as the reference database [4,5]. The main parameters for the assessment of gut microbiota structure and functionality were derived from literature sources (PubMed, Medline, Web of Science) and the Human Microbiome Project database [6], identifying common ranges of relative abundances of key microbial groups related to positive and negative health effects and the production of beneficial metabolites (i.e., short chain fatty acids production SCFA) (Table S1) [7–11].

Table S1. Compositional and functional characteristics of key microbial groups selected to characterize the gut microbiota state, including fixed ranges for relative proportions and typical concentrations of major short-chain fatty acids (SCFAs) produced.

Microbiota group typical abundances in healthy gut	'Normal' abundances of taxa associated with positive health effects	Taxa in higher-than-usual abundances leading to negative health effects	Microbial metabolites
Firmicutes phylum (35.1 - 63.7%)	Christensenellaceae (1.2-7.9%)	Enterococcaceae (0.0-0.12%)	Acetic acid (45 - 120 μmol/g feces)
Bacteroidetes phylum (16.5 - 35.0%)	Faecalibacterium (5-18%)	Enterobacteriaceae (2.3-5.7%)	Butyric acid (8 - 24 μmol/g feces)
Proteobacteria phylum (0.6 - 9.0%)	Akkermansia (3.0-6.4%)	Streptococcus (0.1-2.5%)	Propionic acid (30 -44 μmol/g feces)
Actinobacteria phylum (1.1 - 10.3%)	Bifidobacterium (1.2-7.4%)	Klebsiella (0.0-0.17%)	Total SCFAs (50 - 200 μmol/g feces)
Fim/Bact ratio (0.8 – 3.0%)	Lactobacillus (0.01-0.2)	Fusobacterium (0.0-0.005%)	
α-diversity values Observed Species (200 -300) Shannon index (3.4 - 4.4)	Clostridium IVXa Group (2.5-14.5%)		

Supplementary References

1. BIOTOOLS B&M Labs. *SPEEDTOOLS TISSUE DNA EXTRACTION KIT: Kit Para La Extracción y Purificación de ADN Genómico a Partir de Tejidos, Células, Bacterias, Hongos y Fluidos Orgánicos. Instrucciones de Uso*. Ref. 21.136/7.; 2015. Accessed April 10, 2024. http://www.biotools.eu/documentospdf/Speedtools_Tissue_DNA_Extraction_Kit.esp.ed07.Mayo15.pdf

2. ThermoFisher Scientific. *QuantStudio 12K Flex Real–Time PCR System v1.4 Maintenance and Administration Guide*. 4470689 Rev. D. (ThermoFisher Scientific, ed.).; 2022. Accessed April 10, 2024. https://assets.thermofisher.com/TFS-Assets/LSG/manuals/4470689_QS12K_Flex_Maint_Admin_UG.pdf

3. Molinero N, Taladrid D, Zorraquín-Peña I, et al. Ulcerative Colitis Seems to Imply Oral Microbiome Dysbiosis. *Curr Issues Mol Biol*. 2022;44(4):1513-1527. doi:10.3390/cimb44040103

4. Callahan BJ, McMurdie PJ, Rosen MJ, Han AW, Johnson AJA, Holmes SP. DADA2: High-resolution sample inference from Illumina amplicon data. *Nat Methods*. 2016;13(7):581-583. doi:10.1038/nmeth.3869

5. Quast C, Pruesse E, Yilmaz P, et al. The SILVA ribosomal RNA gene database project: improved data processing and web-based tools. *Nucleic Acids Res*. 2012;41(D1):D590-D596. doi:10.1093/nar/gks1219

6. University of Chicago and the Ontario Institute of Cancer Research (OICR). NIH Human Microbiome Project. Accessed December 2, 2024. https://hmpdacc.org/

7. Taladrid D, de Celis M, Belda I, Bartolomé B, Moreno-Arribas MV. Hypertension- and glycaemia-lowering effects of a grape-pomace-derived seasoning in high-cardiovascular risk and healthy subjects. Interplay with the gut microbiome. *Food Funct*. 2022;13(4):2068-2082. doi:10.1039/D1FO03942C

8. Almeida A, Mitchell AL, Boland M, et al. A new genomic blueprint of the human gut microbiota. *Nature*. 2019;568(7753):499-504. doi:10.1038/s41586-019-0965-1

9. Rinninella E, Raoul P, Cintoni M, et al. What is the Healthy Gut Microbiota Composition? A Changing Ecosystem across Age, Environment, Diet, and Diseases. *Microorganisms*. 2019;7(1):14. doi:10.3390/microorganisms7010014

10. Latorre-Pérez A, Hernández M, Iglesias JR, et al. The Spanish gut microbiome reveals links between microorganisms and Mediterranean diet. *Sci Rep*. 2021;11(1):21602. doi:10.1038/s41598-021-01002-1

11. Casén C, Vebø HC, Sekelja M, et al. Deviations in human gut microbiota: a novel diagnostic test for determining dysbiosis in patients with IBS or IBD. *Aliment Pharmacol Ther*. 2015;42(1):71-83. doi:10.1111/apt.13236
