# Supplementary material for: Evaluation of the ALIBIRD mHealth Platform for Care of Patients With Lung Cancer: Prospective Pilot Study
Source: JMIR Cancer. 2026 Feb 11;12:e69525. doi: 10.2196/69525 (PMC12893646; doi:10.2196/69525)
Supplement: Multimedia Appendix 4 [file cancer-v12-e69525-s004.pdf]

Multimedia Appendix 4: **Detailed Nutrigenetic Results.** This appendix provides a detailed summary of the nutrigenetic results obtained from the 20 participants enrolled at baseline. Genetic testing targeted 20 variants associated with lifestyle habits, nutrition, and cancer-related factors. *Figures and tables are numbered starting from S1 within each Multimedia Appendix.*

Table S1 presents the distribution of favorable, neutral, and unfavorable genotypes for each variant or gene group, categorized by biological function: biorhythm, food intolerance, response to compounds, and oncology-relevant parameters. These data were used to generate the composite scores that guided the personalized nutritional recommendations delivered to participants through the ALIBIRD mobile app.

Table S1. Detailed results of nutrigenetic testing, showing the distribution of favorable, neutral, and unfavorable variants across all study participants (N=20). The results are categorized into groups associated with lifestyle habits, nutrition, and cancer-related factors.

	Analyzed genes	Favorable N (%)	Neutral N (%)	Unfavorable N (%)
**Biorhythm**				
Predisposition to sleepiness	CLOCK[a]	12 (60)	7 (35)	1 (5)
Physiological rhythms	CLOCK[a]	12 (60)	NC[v]	8 (40)
**Food intolerance**				
Gluten tolerance	HLA-DQA1[b]; HLA-DRA[c]; HLA-DQB1[d]; HLA-DQ[e]	16 (80)	4 (20)	0 (0)
Lactose metabolism	MCM6[f]	15 (75)	NC[v]	5 (25)
**Response to compounds**				
Gluten tolerance	HLA-DQA1[b]; HLA-DRA[c]; HLA-DQB1[d]; HLA-DQ[e]	16 (80)	4 (20)	0 (0)
Lactose metabolism	MCM6[f]	15 (75)	NC[v]	5 (25)
**Response to compounds**				
Caffein	CYP1A[g]	11 (55)	7 (35)	2 (10)
Tobacco	CYP2A6[h]	2 (10)	9 (45)	6 (45)
Alcohol	ALDH2[i]	13 (68)	6 (32)	0
**Parameters relevant for oncology**				
Systemic inflammation	IL-6[j]; TNF-a[k]; GCKR[l]; LPL[m]	5 (25)	14 (70)	1 (5)
Energetic metabolism	MC4R[n]; UCP2[o]	9 (45)	6 (30)	5 (25)
Apetite	GHRL[p]	6 (30)	0	14 (70)
Lipid metabolism	ABCA1[q]<br>SCD[r,s]<br>ACSL1[t]	10 (50)<br>17 (85)<br>18(90)	NC[v]	10 (50)<br>2 (10)<br>2 (10)
Statins	SLCO1B1[u]	15(75)	4 (20)	1 (5)

[a]CLOCK: clock circadian regulator.
[b]HLA-DQA1: major histocompatibility complex, class II, DQ alpha.
[c]HLA-DRA: major histocompatibility complex, class II, DR alpha.
[d]HLA-DQB1: major histocompatibility complex, DQ .
[e]HLA-DQ: major histocompatibility complex, class II, DQ beta 1
[f]MCM6: minichromosome maintenance complex component 6.

[g]CYP1A1: cytochrome P450 family 1 subfamily A member 1
[h]CYP2A6: cytochrome P450 family 2 subfamily A member 6.
[i]ALDH2: aldehyde dehydrogenase 2 family member.
[j]IL-6: interleukin 6.
[k]TNF-a: tumor necrosis factor alpha.
[l]GCKR: glucokinase regulator.
[m]LPL: lipoprotein lipase
[n]MC4R: melanocortin 4 receptor.
[o]UCP2: uncoupling protein 2.
[p]GHRL: ghrelin and obestatin prepropeptide.
[q]ABCA1: ATP binding cassette subfamily A member 1.
[r]SCD: stearoyl-CoA desaturase.
[s]One result was considered invalid, resulting in a reduced sample size of 19.
[t]ACSL1: acyl-CoA synthetase long chain family member 1.
[u]SLCO1B1: solute carrier organic anion transporter family member 1B1
[v]NC: Not contemplated.
